# Noradrenergic Blockade of Memory Reconsolidation: A Failure to Reduce Conditioned Fear Responding

**DOI:** 10.3389/fnbeh.2014.00412

**Published:** 2014-11-28

**Authors:** Marieke Geerte Nynke Bos, Tom Beckers, Merel Kindt

**Affiliations:** ^1^Department of Clinical Psychology, University of Amsterdam, Amsterdam, Netherlands; ^2^Amsterdam Brain and Cognition, University of Amsterdam, Amsterdam, Netherlands; ^3^Department of Psychology, KU Leuven, Leuven, Belgium

**Keywords:** reconsolidation, extinction learning, fear conditioning, fear potentiated startle, propranolol

## Abstract

Upon recall, a memory can enter a labile state in which it requires new protein synthesis to restabilize. This two-phased reconsolidation process raises the prospect to directly target excessive fear memory as opposed to the formation of inhibitory memory following extinction training. In our previous studies, we convincingly demonstrated that 40 mg propranolol HCl administration *before* or *after* memory reactivation eliminated the emotional expression of fear memory indexed by the fear potentiated startle reflex. To apply this procedure in clinical practice it is important to understand the optimal and boundary conditions of this procedure. As part of a large project aimed at unraveling putative boundary conditions of disrupting reconsolidation of associative fear memory with propranolol HCl, we again tested our memory reconsolidation procedure. Participants (*N* = 44) underwent a three-day differential fear conditioning procedure. Twenty-four hours after fear acquisition, participants received 40 mg propranolol HCl prior to memory reactivation. The next day, participants were subjected to extinction training and reinstatement testing. In sharp contrast to our previous findings, propranolol HCl before memory reactivation did not attenuate the startle fear response. Remarkably, the startle fear response even persisted during extinction training and did not show the usually observed gradual decline in conditioned physiological responding (startle potentiation and skin conductance) upon repeated unreinforced trials. We discuss these unexpected findings and propose some potential explanations. It remains, however, unclear why we observed a resistance to reduce conditioned fear responding by either disrupting reconsolidation or extinction training. The present results underscore that the success of human fear conditioning research may depend on subtle manipulations and instructions.

## Introduction

Reconsolidation has attracted much attention in the literature because it provides the opportunity to weaken excessive fear memory with the promise to permanently reduce previously learned fear responding. Reconsolidation refers to the process wherein a memory trace, following retrieval, enters a temporarily labile state requiring *de novo* protein synthesis for restabilization (Nader et al., 2000; Sara, 2000). The process of reconsolidation is typically demonstrated in animals through the amnestic effects of protein synthesis inhibitors administered after memory reactivation (e.g., Nader et al., 2000; Sara, 2000). In a series of human fear conditioning studies in our lab, we convincingly demonstrated that administration of the β-adrenergic receptor antagonist propranolol HCl *before* or *after* memory reactivation eliminated the emotional expression of fear memory (i.e., fear potentiated startle reflex), while leaving the declarative memory intact (Kindt et al., 2009; Soeter and Kindt, 2010, 2011a, 2012a,b; Sevenster et al., 2012, 2013, 2014a). Importantly, this fear-reducing effect was long-lasting (Soeter and Kindt, 2010) and generalized to semantically related stimuli (Soeter and Kindt, 2011b, 2012b) as well as to other contexts (Soeter and Kindt, 2012a). These findings mark the potential of disrupting reconsolidation with propranolol to weaken and perhaps even erase a previously learned fear response.

Reconsolidation has been shown across several species and with different protocols, which underlines the robustness of this phenomenon. However, there are also certain parameters – so-called boundary conditions – that may constrain reconsolidation to occur (Nader and Hardt, 2009). Recently, it has been shown that mere retrieval is not sufficient to render a memory trace labile (Pedreira et al., 2004; Sevenster et al., 2012, 2013, 2014a; Díaz-Mataix et al., 2013). A prerequisite to trigger reconsolidation is the experience of a prediction error upon retrieval, which refers to a mismatch between what is expected and the actual experience. Other putative boundary conditions that have been proposed are the strength (Suzuki et al., 2004; Wang et al., 2009, but see Soeter and Kindt, 2012b) and the age (Milekic and Alberini, 2002; Suzuki et al., 2004) of the consolidated memory trace, repetitive or prolonged memory reactivation (i.e., extinction learning) (e.g., Eisenberg et al., 2003; Bos et al., 2012; Sevenster et al., 2014a) and the spatial context during memory reactivation (Hupbach et al., 2008). Thus, memory reconsolidation upon retrieval may only occur under the appropriate conditions.

The effectiveness of disrupting memory reconsolidation seems to be sensitive to individual differences as well, such as genetic polymorphisms (Agren et al., 2012) and trait anxiety (Soeter and Kindt, 2013). Collapsing the data of most of our previous experiments revealed that individuals that can be characterized by high levels of trait anxiety showed somewhat less fear reduction following propranolol HCl administration *before* or *after* memory reactivation. This may indicate that for individuals high in trait anxiety either another reactivation procedure or higher doses of propranolol HCl are required to trigger or interfere with the process of fear memory reconsolidation (Soeter and Kindt, 2013).

The ability to eliminate the emotional expression of fear memory by disrupting memory reconsolidation may substantially enhance treatment efficacy in the near future. To apply this procedure in clinical practice, it is, however, essential to understand the optimal and boundary conditions of this procedure. The reported data in this article were part of a larger project aimed at unraveling putative boundary conditions of disrupting reconsolidation of memory in humans with propranolol HCl. The project started with pilot studies to demonstrate our reconsolidation effect, but turned out in a failure to replicate our previous findings. Along the way, we made subtle changes in our instructions in order to optimize the procedure. It bears mentioning that we have replicated our original finding of disrupting fear memory reconsolidation (Kindt et al., 2009) multiple times (Soeter and Kindt, 2010, 2011a, 2012a,b; Sevenster et al., 2012, 2013, 2014a). Hence, the initial objective of the current study was not to demonstrate another replication. Nevertheless, presenting these unexpected results is important as they might at least prevent a publication bias, even though it remains unclear why we failed to reduce the conditioned fear response (CR).

We report the data of 44 participants who underwent a three-day differential fear conditioning paradigm including the following phases: acquisition (day 1), memory reactivation (day 2), differential extinction training, and reinstatement testing (day 3). On day 1, participants were differentially conditioned by pairing one picture of a spider (conditioned stimulus; CS1) with an aversive electric shock (unconditioned stimulus; US), while another picture of a spider was never paired with the US. On day 2, participants received propranolol HCl 90 min before the fear memory was reactivated by a non-reinforced presentation of the feared stimulus (CS1-R). Memory retention of the CS1 and CS2 was tested 24 h later. At test, participants were exposed to differential extinction training. To investigate the return of fear, three reminder shocks were presented to reinstate the CR. The CR was measured by the fear potentiated startle reflex, skin conductance, and online US-expectancy ratings. Based on our previous findings, we expected that propranolol HCl administration before memory reactivation would diminish the emotional expression of fear memory (i.e., fear potentiated startle reflex) 24 h later, but would leave the declarative memory unaffected (i.e., online US expectancy). Given that skin conductance seems strongly related to US-expectancy ratings in fear conditioning paradigms (e.g., Hamm and Weike, 2005; Soeter and Kindt, 2010; Sevenster et al., 2014b), we expected that propranolol HCl would not affect skin conductance either.

The current results demonstrated that propranolol HCl administration prior to reactivation did not affect the emotional expression of fear. That is, the acquired fear potentiated startle response remained intact on day 3 at test, throughout extinction training and at reinstatement testing. These results are in sharp contrast to previous findings on human fear conditioning and we will discuss possible explanations of these unexpected results.

## Materials and Methods

### Participants

A total of 44 undergraduate students (16 men and 28 women), ranging in age between 18 and 26 years (Mean ± SD age – 20.66 ± 2.17 years) participated in a propranolol HCl condition. All participants were assessed to be free from any current or previous medical or psychiatric condition that would contra-indicate participation [i.e., pregnancy, blood pressure (BP) < 90/60, seizure disorder, respiratory disorder, cardiovascular disease, diabetes, liver/kidney disorder, medication use (other than oral contraceptives), depression, anxiety or psychosis]. Furthermore, participants who scored above 26 on the anxiety sensitivity index (ASI, Peterson and Reiss, 1992) were excluded from participation, as they might experience difficulty with any temporary symptoms induced by propranolol HCl. Participants received either course credits or were paid a small amount of money (€35) for their participation. The ethical committee of the University of Amsterdam approved all procedures and informed consent was obtained from all participants.

### Measurements and apparatus

#### Stimuli

The conditioned stimuli (CS) consisted of two different pictures of spiders (IAPS, nr 1200 and 1201, Lang et al., 2005). Assignment of the slides to CS1 + and CS2− was counterbalanced across participants. An electric stimulus of 2 ms served as the US. The electric stimulus was delivered through a pair of Ag electrodes of 20 by 25 mm with a fixed inter-electrode mid-distance of 45 mm, which were controlled by a Digitimer DS7A constant current stimulator (Hertfordshire, UK). A conductive gel (Signa, Parker Laboratories) was applied between the electrodes and the skin.

#### Fear potentiated startle reflex

The acoustic startle reflex was used as an index of the emotional expression of the CR. Potentiation of the acoustic startle reflex to a loud noise was measured by electromyography (EMG) of the right orbicularis oculi muscle. Two 7 mm Ag/AgCl electrodes filled with electrolyte gel were attached approximately 1 cm under the pupil and 1 cm below the lateral canthus, respectively; a ground electrode was placed on the forehead (Blumenthal et al., 2005). The acoustic stimulus was a burst of white noise (40 ms; 104 dB) with near-instantaneous rise time. Startle probe and background noise were both presented binaurally over headphones (Sennheiser 25 I-II). The eyeblink EMG activity was measured using a bundled pairs of electrode wires connected to a front-end amplifier with an input resistance of 10 MΩ and a bandwidth of DC-1500 Hz. The EMG signal was sampled at 1000 Hz. Note that in our first study on reconsolidation (Kindt et al., 2009) the magnitude of the startle response was multiplied by factor two.

#### Skin conductance response

Electrodermal activity was measured using an input device with a peak–peak sine shaped excitation voltage (± 0.5 V) of 50 Hz. Two Ag/AgCl electrodes of 20 by 16 mm were attached to the medial phalanx surfaces of the middle and fourth finger of the non-dominant hand. The signal from the input device was led through a signal-conditioning amplifier and the analog output was digitized at 1000 Hz by a 16-bit AD-converter (National Instruments, NI-6224).

#### US-expectancy ratings

US-expectancy ratings were used as an index of the cognitive expression of the CR. US expectancy was measured online during the first 7 s of each CS presentation on an 11-point visual analog scale (VAS) ranging from −5 (certainly no shock) through 0 (uncertain) to 5 (certainly a shock). The scale was continuously presented at the bottom of the computer screen in order to focus participants’ attention to the CS–US contingencies. Participants rated US-expectancy levels by shifting the cursor on the scale with the mouse and confirmed their ratings by clicking the left mouse button.

#### US-evaluation

Evaluation of the US was assessed on an 11-point VAS scale ranging from −5 (unpleasant) to 5 (pleasant).

#### Anxiety assessment

The spider phobia questionnaire (SPQ, Klorman et al., 1974) was used to determine the degree of spider fear. The ASI was used to assess participants’ tendency to respond anxiously to potential temporary symptoms of the use of propranolol HCl. State and trait anxiety were measured with the State and Trait Anxiety Inventory (STAI-S and STAI-T, Spielberger, 1970). State anxiety was used to assess the influence of propranolol HCl. The spider fear (SPQ) and trait anxiety (STAI-T) were measured to explore the effect of individual differences on disrupting reconsolidation of spider fear memory.

#### Pharmacological treatment

Propranolol HCl (40 mg) pills were prepared by a pharmacy (Huygens Apotheek, Voorburg). To assess whether propranolol HCl exerted its physiological effect, we measured BP using an electronic sphygmomanometer, with the cuff attached to the right upper arm.

### Experimental design and procedure

**Figure 1:**
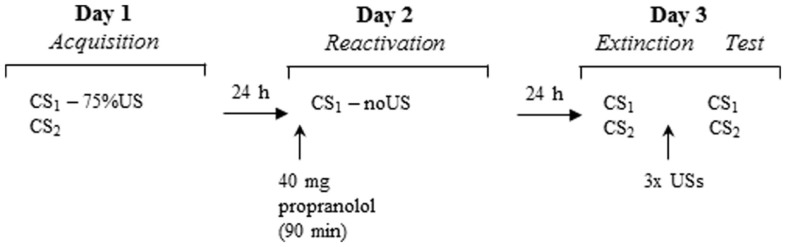
**Schematic of the experimental design over three days**.

Participants underwent a differential fear conditioning procedure that consisted of several phases across three consecutive days (see Figure 1). Each testing session started with 10 startle habituation trials to stabilize baseline startle reactivity. A 70-dB broadband noise was used as a background noise throughout all sessions. Testing procedures were similar to the study of Kindt et al. (2009) unless otherwise noted.

#### Day 1 – acquisition

Prior to the experimental procedure, participants were interviewed regarding their medical or psychiatric condition. Thereafter ASI and SPQ were administered. After attachment of the EMG, SCR, and shock electrodes, individual shock intensity was determined. The work-up procedure started at a very mild level of shock (1 mA) and gradually increased until the shock level reached a level that the participant determined as uncomfortable, but not painful. The intensity of the shock remained the same throughout the experiment. After US selection, participants were instructed regarding the CS. Participants received the instruction to look carefully at the slides, of which one would in most cases be followed by shock and the other would never be followed by shock. They were instructed to rate their expectancy of the shock during each slide. During *acquisition*, the CS1 and CS2 stimuli were presented eight times for 8 s. The startle probe was presented 7 s after stimulus onset. To delay extinction learning the subsequent days, a fixed partial reinforcement schedule was applied (first and fifth CS1 trial were unreinforced). The US was presented 7.5 s after stimulus onset. To assess fear responses to the context, eight baseline startle probes were presented during the inter-trial intervals (ITI; Noise Alone trials, NA). Mean ITI was 20 s (range: 15–25). Trial order and ITI were semi-random, with the restriction that no more than two consecutive trials or ITI durations were of the same type. Characteristics of the CS, ITI and trial order were similar for the following days. At the end of session one, participants evaluated the US and CS.

#### Day 2 – memory reactivation

Blood pressure and STAI-S were assessed before participants received an oral dose of 40 mg of propranolol HCl (single blind). In view of the kinetics of the peak plasma concentration (1–2 h) of propranolol HCl (Gilman and Goodman, 1996), the memory reactivation procedure started 90 min after pill intake. After attachment of the electrodes, participants were instructed that the experiment would be a continuation of the previous day and that they had to remember what they learned the day before. The first 24 participants received the same instruction as in our first reconsolidation study (Kindt et al., 2009): one stimulus would sometimes be followed by the shock and the other stimulus would never be followed by a shock. The explicit instruction regarding the control stimulus (CS2−) was given to reduce the ambiguity of this stimulus during the subsequent testing days and to ensure that participants did not expect a reversed reinforcement schedule. Given that we failed to replicate our reconsolidation finding in this sample (*n* = 24), we changed our instructions. To increase the likelihood that participants experienced a discrepancy between the expectancy of the shock and the absence of receiving the shock as was triggered by the retrieval trial, we told the subsequent sample of participants (*n* = 20) that one stimulus would *in most cases* be followed by the shock and the other stimulus would never be followed by the shock. The *memory reactivation* phase consisted of 1 unreinforced CS1 trial, and 1 NA trial. BP and STAI-S were again assessed after memory reactivation.

#### Day 3 – extinction and reinstatement

Upon arrival at the lab, BP was again assessed. After attachment of the electrodes, participants were only informed that the same stimuli were presented as on day 1. The *differential extinction* phase consisted of randomized presentations of unreinforced CS1, CS2, and NA trials (number of presentations was 12 or 16). After the extinction procedure, participants received three unsignaled shocks. The time between extinction and the reinstatement shocks was 19 s, the time between the shocks was on average 28 s. The three unsignaled shocks were followed by *reinstatement testing*, which consisted of randomized and unreinforced presentations of CS1, CS2, and NA (number of presentations was 5 or 6). At the end of the experiment, participants completed the STAI-T and STAI-S.

### Data reduction and statistical analysis

All physiological data were processed with VSRRP 98 v9.0 (developed by Technical Support Group UvA Psychology). For the startle response data, an analog notch filter was set at 50 Hz to remove interference of the mains noise. The raw EMG signal was amplified and band-pass filtered (28–500 Hz butterworth forth order) (Van Boxtel et al., 1998; Blumenthal et al., 2005). Startle magnitude was defined as the amplitude of the first peak within a 20–200 ms interval following the startle probe onset. Outliers were determined over all trials (−3 < *Z* > 3; 0.86%) and individually replaced by the linear trend of that data point for each phase and CS type separately.

Electrodermal responses elicited by the CS were determined by taking the baseline (i.e., 2 s before CS onset) to peak difference within the 7 s window following stimulus onset. Outliers were determined over all trials (−3 < *Z* > 3; 0.69%) and replaced following the same procedure as the startle data. To increase normality, raw SCR were log-transformed (LG10(SCR + 1.5) (Fowles et al., 1981). Due to technical problems with the skin conductance electrodes, we did not obtain proper data for six participants and those participants were discarded from analyses.

US-expectancy ratings were multiplied by 20 to create a scale from −100 to 100.

Statistical analyses were performed using the SPSS statistical software package (SPSS Inc., Chicago, IL, USA). Given that we found no differences between the two instruction groups (*n* = 20; *n* = 24), we collapsed the data and described the analyses over all participants. Repeated measures analyses of variance (ANOVA) were performed for each dependent variable separately to test for acquisition, extinction and reinstatement effects. Stimulus (CS1 + ∕− versus CS2−) and trial (all trials within each phase and the first and last trial between phases) were used as the within-subject factors. When the assumption of sphericity was violated, Huynh–Feldt correction was applied when the epsilon was ≥0.75, otherwise the multivariate data were interpreted (Field, 2013).

Next, we performed a hierarchical regression analysis to explore whether vulnerability factors, like trait anxiety, SPQ, or responsiveness to propranolol HCl (decrease in BP) could predict individual differences among participants on (1) change in differential expression of fear from day 1 to day 3 and (2) differential expression of fear at reinstatement test. To obtain a single value for the change in fear expression due to propranolol HCl administration before memory reactivation, we subtracted the differential startle response of the last trial of acquisition (a8; CS1+ minus CS2−) from the differential startle response of the first trial of extinction training (e1; CS1− minus CS2−). Furthermore, we calculated the differential startle response at the first reinstatement test trial (r1; CS1− minus CS2−). Based on our previous study (Soeter and Kindt, 2013), we first entered trait anxiety as potential predictor in the hierarchical regression model (first level) followed by trait anxiety, state anxiety, SPQ and percentage change in systolic and diastolic BP (second level).

An alpha level of.05 was used for all statistical tests.

## Results

### Anxiety assessment and US characteristics

Table 1 presents the mean (SD) scores on the anxiety questionnaires and characteristics of the US. Shock intensity ranged from 4 to 40 mA and was negatively rated by participants (range: 0 to −5).

**Table 1:** **Mean values ± SEM of reported spider fear (SPQ), trait anxiety, anxiety sensitivity (ASI), intensity of the US and subjective evaluation of the US**.

**Anxiety assessment**	
Spider fear	6.77 ± 0.88
Trait anxiety	34.11 ± 1.16
Anxiety sensitivity	9.80 ± 0.82
**US characteristics**	
Shock intensity (mA)	17.91 ± 1.44
Shock evaluation	−3.16 ± 0.15

### Manipulation check of propranolol HCl

Analysis of the effect of propranolol HCl on BP demonstrated the expected decrease in both systolic (*t*(43) = 10.54, *p* < 0.001, *d* = 1.59) and diastolic BP (*t*(43) = 4.89, *p* < 0.001, *d* = 1.27) from baseline to 95 min after pill intake. Systolic BP dropped from 120.36 (SD = 11.38) to 106.93 (SD = 10.95) and diastolic BP from 69.07 (SD = 4.92) to 65.18 (SD = 6.15). This suggests that propranolol HCl manipulation exerted its intended physiological effect. In line with previous studies (e.g., Grillon et al., 2004; Soeter and Kindt, 2010), propranolol HCl did not affect levels of state anxiety (*t* < 1.0).

### Fear potentiated startle reflex

**Figure 2:**
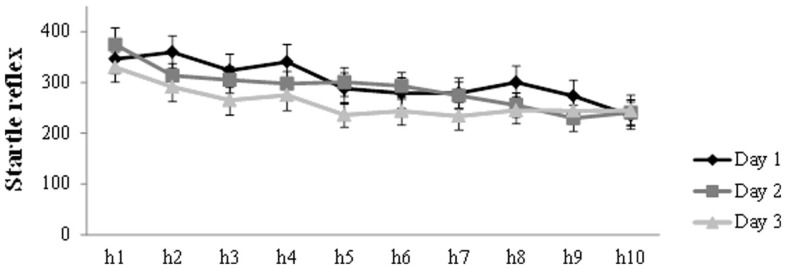
**Mean startle potentiation to the 10 habituation trials prior to acquisition (day 1), reactivation (day 2), and differential extinction training (day 3)**. Error bars represent SEM.

Figure 2 presents the general decrease in startle amplitude during the habituation trials at the start of each session. Analysis on the habituation trials (h1–h10) for the three days, showed a main effect of Trial (*F*(6.22, 267.28) = 15.61, *p* < 0.001, η_p_^2^ = 0.27) and Day (*F*(2,86) = 4.96, *p* = 0.009, η_p_^2^ = 0.10), in the absence of a Trial by Day interaction. There was a general decrease in startle amplitude during habituation on each day. Furthermore, simple contrasts revealed that startle amplitude was lower on day 3 compared to day 1 (*F*(1,43) = 7.73, *p* = 0.008, η_p_^2^ = 0.15) and day 2 (*F*(1,43) = 5.30, *p* = 0.026, η_p_^2^ = 0.11).

**Figure 3:**
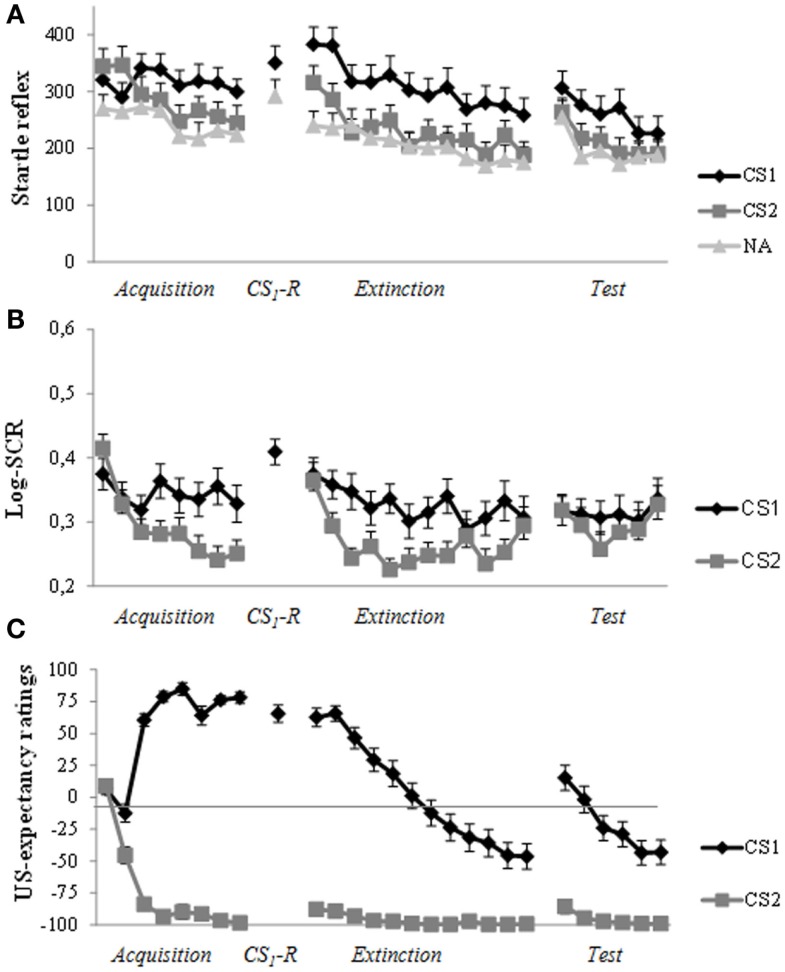
**Startle reflex, skin conductance and US-expectancy ratings during acquisition (day 1), memory reactivation (day 2), extinction training, and reinstatement testing (day 3)**. **(A)** Successful acquisition for the fear potentiated startle reflex. Unexpectedly, propranolol HCl administration prior to memory reactivation did not attenuate the startle fear response at day 3. The differential startle response remained during extinction training and reinstatement test. **(B)** Robust acquisition for SCR. There was a strong increase in response to the control stimulus (CS2) at the start of extinction training (day 3). The three reminder shocks did not reinstate a differential response. **(C)** The predicted pattern of acquisition, extinction, and reinstatement for US-expectancy ratings. Error bars represent standard error to the mean (SEM).

As can be seen in Figure 3A, robust levels of fear acquisition were obtained, as indicated by a gradual increase (trial a1–a8) in differentiation between CS1 and CS2 (Stimulus × Trial: *F*(5.94,255.28) = 4.19, *p* = 0.001, η_p_^2^ = 0.09). At memory reactivation, we observed stronger startle amplitude in response to CS1 compared to NA (*t*(43) = 2.51, *p* = 0.016, *d* = 0.38). In sharp contrast to previous studies of our lab, administration of propranolol HCl did not result in a decrease in differential startle response from the end of day 1 (a8) to the first extinction trial (e1) 48 h later (Stimulus × Trial: *F* < 1.0). As can be seen in Figure 3A, the differentiation in startle amplitude between CS1 and CS2 remained (stimulus: *F*(1,43) = 9.00, *p* = 0.004, η_p_^2^ = 0.17). Moreover, there was a general increase in startle reactivity from day 1 to day 3 (Trial: *F*(1,43) = 11.34, *p* = 0.002, η_p_^2^ = 0.21). Remarkably, analysis of differential extinction training on day 3 showed no decrease in differential startle responding either (trial 1–12; Stimulus × Trial: *F* < 1.0; Stimulus: *F*(1,43) = 20.43, *p* < 0.001, η_p_^2^ = 0.32). There was, however, a general decrease in startle reactivity during extinction training (Trial: *F*(6.85, 294,71) = 11.28, *p* < 0.001, η_p_^2^ = 0.21). Additional analysis on the last extinction trial (e12) confirmed that extinction training was unsuccessful, as indicated by a difference in startle amplitude between CS1 and CS2 (*t*(43) = 2.77, *p* = 0.008, *d* = 0.43). Given that the differential startle response did not diminish after extinction training, the three unsignaled shocks could not properly reinstate the differential startle response from the end of extinction (e12) to the first reinstatement test trial (CS × Trial: *F*(1,42) = 1.05, *p* = 0.31; CS: *F*(1,42) = 6.20, *p* = 0.017, η_p_^2^ = 0.13). Nevertheless, there was a general increase in startle amplitude (Trial: *F*(1,42) = 11.04, *p* = 0.002, η_p_^2^ = 0.21). Re-extinction learning on day 3 did also not diminish differentiation in startle amplitude between CS1 and CS2 (trial r1–r6; Stimulus × Trial: *F* < 1.0; Stimulus: *F* (1,42) = 7.31, *p* = 0.01, η_p_^2^ = 0.15). Again, there was only a decrease in general startle reactivity during re-extinction training (Trial: *F*(4.23,177.58) = 6.88, *p* < 0.001, η_p_^2^ = 0.14).

### Skin conductance response

In line with our previous findings, administration of propranolol HCl prior to memory reactivation did not exert any fear-reducing effect on skin conductance responding. As can be seen in Figure 3B, differential SCR was observed from trial 1 to trial 8 (Stimulus × Trial: *F*(7, 259) = 3.37, *p* = 0.002, η_p_^2^ = 0.08). In line with our previous studies, the differential response remained from the end of acquisition (a8) to the start of extinction training (e1) (Stimulus: *F*(1,37) = 4.28, *p* = 0.046, η_p_^2^ = 0.10). Moreover, there was a slight differential increase in SCR from the end of day 1 (a8) to the start of day 3 (e1; Stimulus × Trial: *F*(1,37) = 3.80, *p* = 0.059, η_p_^2^ = 0.09; Trial: *F*(1,37) = 16.53, *p* < 0.001, η_p_^2^ = 0.31). As can be seen in Figure 3B, there was a strong increase to CS2 (*t*(37) = 4.57, *p* < 0.001, *d* = 0.77). Analysis of extinction training (trial e1–e12) showed a main effect of Stimulus (*F*(1,37) = 25.38, *p* < 0.001, η_p_^2^ = 0.41) and Trial (*F*(7.32, 270.72) = 5.07, *p* < 0.001, η_p_^2^ = 0.12) and a marginally significant Stimulus by Trial interaction (*F*(7.16, 265.01) = 2.00, *p* = 0.054, η_p_^2^ = 0.05). The three unsignaled shocks did not reinstate the differential SCR (Stimulus: *F* < 1.0; Trial: *F* < 1.0; Stimulus × Trial: *F* < 1.0).

### US-expectancy ratings

Figure 3C displays the mean US-expectancy ratings for CS1 and CS2 across trials. Differential US-expectancy ratings were acquired from trial 1 to 8 (Stimulus × Trial: *F*(7,37) = 97.06, *p* < 0.001, η_p_^2^ = 0.95). Differentiation in US-expectancy ratings diminished from the end of acquisition (a8) to the start of extinction training 48 h later (Stimulus × Trial: *F* (1,43) = 7.51, *p* = 0.009, η_p_^2^ = 0.15). Planned comparisons of Time revealed that US-expectancy ratings of CS1 remained relatively stable (Trial: *F*(1,43) = 3.25, *p* = 0.078, η_p_^2^ = 0.07), whereas US-expectancy ratings of CS2 increased from the end of acquisition to the start of extinction (Trial: *F*(1,43) = 7.31, *p* = 0.01, η_p_^2^ = 0.15). Analysis of extinction training showed that there was a strong decrease in differential US-expectancy rating across trials (trial: e1–e12; Stimulus × Trial: *F*(11,33) = 12.91, *p* < 0.001, η_p_^2^ = 0.81). The three unsignaled shocks reinstated the expectancy of the US from the end of extinction training (e12) to the first reinstatement test trial (Stimulus × Trial: *F* (1,43) = 21.81, *p* < 0.001, η_p_^2^ = 0.34). Furthermore, re-extinction learning diminished the differential US-expectancy rating (Stimulus × Trial: *F*(5,39) = 10.39, *p* < 0.001, η_p_^2^ = 0.57).

### Hierarchical regression analysis

As can be seen in Table 2, we did not identify significant predictors that could explain individual variability in the persistence of differential startle responding from day 1 (a8) to day 3 (e1). Furthermore, we found no predictors of differential reinstatement at trial 1 (r1).

**Table 2:** **Results from the hierarchical regression analyses**.

	Differential fear response day 1 to day 3		Differential fear response at reinstatement test	
	β	*t*	β	*t*
**Step I**				
Constant	108.59	<1	−7.13	<1
STAI-T	−2.83 (−10.44, 4.79)	<1	1.43 (−5.05, 7.92)	<1
**Step II**				
Constant	146.66	<1	−28.99	<1
STAI-T	−2.26 (−11.82, 7.31)	<1	2.89 (−4.99, 10.77)	<1
SPQ	4.03 (−6.82, 14.87)	<1	5.08 (−14.25, 4.09)	1.12
STAI-S	−2.50 (−10.57, 5.57)	<1	−1.83 (−8.67, 5.01)	<1
Systolic BP	−1.31 (−10.72, 8.11)	<1	−5.19 (−13.05, 2.67)	1.34
Diastolic BP	2.93 (−5.27, 11.12)	<1	−1.66 (−8.41, 5.10)	<1

*The parameters predicted neither fear retention (day 1 versus day 3) nor the differential fear response at reinstatement test. Unstandardized β coefficients (95% confidence intervals) and *t* scores are presented*.

## Discussion

In sharp contrast to our previous findings, administration of a single dose of 40 mg propranolol HCl prior to memory reactivation did not attenuate the emotional expression of fear 24 h later. It should be noted, however, that the conditioned fear responding (i.e., startle potentiation and SCR) did also not decline during extinction training. Given that this study was highly similar to the experimental condition of our first study on disrupting memory reconsolidation (Kindt et al., 2009), the current results cannot simply be explained by differences in experimental set-up, nor could we identify predictors that were related to the strength of fear memory retention on day 3. Hence, the current findings are puzzling and we can only speculate about possible explanations for the failure to reduce conditioned fear responding.

The current findings clearly indicate that we did not trigger reconsolidation during memory reactivation, given that the emotional expression of fear was not reduced one day later. It has been suggested that a prediction error is a prerequisite for memory reconsolidation, which is triggered by a mismatch between what is expected and what actually happens (i.e., prediction error) upon memory retrieval (Pedreira et al., 2004; Forcato et al., 2009, 2010; Lee, 2009; Sevenster et al., 2012, 2013; Díaz-Mataix et al., 2013). Until recently, the occurrence of prediction error could only be inferred from the observation of the reconsolidation process itself. Previously, we have shown the utility of changes in threat expectancy ratings during memory retrieval as a measure of prediction error that is independent from the occurrence of reconsolidation (Sevenster et al., 2013). The experience of a prediction error upon reactivation critically depends on the interaction between the original learning of the fear association and the memory retrieval. A prediction error may either be triggered by a non-reinforced retrieval trial (CS/no US) or by a reinforced retrieval trial (CS/US) depending on the original learning experience. If memory retrieval follows fully reinforced asymptotic learning episodes, changes in threat expectations from acquisition to test reflect a prediction error. But when memory retrieval follows partially reinforced non-asymptotic learning episodes – such as in several previous studies on reconsolidation (e.g., Kindt et al., 2009; Soeter and Kindt, 2010, 2011a, 2012b) – a shift in threat expectancies is not necessary for post-retrieval plasticity. The current experimental procedure was clearly not designed to explicitly test prediction error driven learning. Therefore, we were unable to assess prediction error independently from the reconsolidation process itself.

A possible explanation for the observed resistance to fear reduction might be a lack of confidence in the CS-US/CS-No US contingencies during the experimental procedure. Even though participants in the current study clearly acquired a differential startle fear response from trial 1 to trial 8, the obtained effect sizes were much smaller than in most of our previous studies (e.g., Kindt et al., 2009; Soeter and Kindt, 2010, 2011b; but, see Sevenster et al., 2012)^1^. Similar to our previous studies, we used a partial reinforcement schedule during acquisition in which 75% of the CS1 trials were reinforced. Thus, it remained – at least to some extent – unpredictable whether the CS was followed by the US. The absence of extinction learning for the fear potentiated startle response and SCR suggests that the participants did not learn from the correcting experience, at least not at the emotional level. The physiological data only showed a non-specific decline of responding, which can be better explained by habituation instead of extinction learning. Even though the US-expectancy ratings showed a general extinction curve, the difference between the ratings of the fear stimulus (CS1) and control stimulus (CS2) remained rather large compared to our previous studies (Kindt et al., 2009; Soeter and Kindt, 2010). This relative resistance to extinction learning suggests a lack of confidence in the non-occurrence of threat. Another observation supporting the idea that our participants did not rely on the experimental procedure is the unexpected increase in US-expectancy ratings to the control stimulus from the end of acquisition to the start of extinction training. This renewed increase in responding to both CS is also shown in the physiological data. There was a strong increase in SCR to the control stimulus (CS2) and a general increase in startle reactivity. This apparent heightened vigilance to the experimental context was not observed in our previous studies (Kindt et al., 2009; Soeter and Kindt, 2010, 2011a, 2012a,b; Sevenster et al., 2012, 2013, 2014a).

These differences in the learned contingencies may explain the current opposing findings. As stated previously, prediction error is a prerequisite to trigger memory reconsolidation and critically depends on the interaction between fear acquisition and memory reactivation (Pedreira et al., 2004; Forcato et al., 2009, 2010; Lee, 2009; Sevenster et al., 2012, 2013; Díaz-Mataix et al., 2013). If the participants were indeed less certain of the contingencies, a single unreinforced retrieval trial may not have been experienced as mismatch. The presentation of the unreinforced CS may therefore have triggered merely retrieval of the previously formed fear association as opposed to memory destabilization (Sevenster et al., 2012, 2013, 2014a). Note that in the study of Sevenster et al. (2012) a similar experimental procedure with a relatively weak fear acquisition (i.e., smaller effect size than the other studies) successfully triggered memory reconsolidation. Though the fear-reducing effect at the retention test was clearly smaller (Sevenster et al., 2012) than in the other reconsolidation studies (Kindt et al., 2009; Soeter and Kindt, 2010, 2011a, 2012a,b; Sevenster et al., 2013). But this is only a *post hoc* explanation for the divergent observations between the current study and our previous findings (Kindt et al., 2009; Soeter and Kindt, 2010, 2011a, 2012a,b; Sevenster et al., 2012, 2013, 2014a), as we infer a lack of prediction error from the weaker contingency learning throughout the experimental phases. Future studies may benefit from protocols that are explicitly designed to assess and manipulate prediction error during memory retrieval to be able to index memory destabilization independently from the effect of reconsolidation (e.g., Sevenster et al., 2013).

Albeit speculative, the question remains why participants in the current experiment would not have had confidence in the experimental procedure. We did not find any predictors that may explain individual differences among participants in the persistence of conditioned fear responding. It has been proposed that differences in trait anxiety and genetic polymorphisms partly determine the effect of disrupting reconsolidation (Agren et al., 2012; Soeter and Kindt, 2013). It is important to note that high trait anxiety was not a boundary condition for disrupting memory reconsolidation and was only related to somewhat less pronounced reduction in fear, but not to the return of fear after the reminder shocks. Furthermore, if we compare participant characteristics between the current study and our original study (Kindt et al., 2009), we did not find any difference in level of trait anxiety, fear of spiders, anxiety sensitivity or subjective evaluation of the shock (all *t*s < 1). Nevertheless, participants in the current study selected higher levels of shock intensity (*M* = 17.91 mA, SD = 9.54) compared to our original study (*M* = 13.85 mA, SD = 6.66, *t*(102) = 2.56, *p* = 0.01), but not compared to the study of Soeter and Kindt (2010) (*M* = 15.52 mA, *SD* = 11.0; *t*(102) < 1), in which a similar protocol was utilized.

A limitation of the current study is the lack of a placebo control condition, which obviously precludes drawing firm conclusions about the observed resistance of fear reduction. We can therefore not exclude the possibility that propranolol HCl before memory reactivation may have affected the fear memory 24 h later to some extent. Because the original objective of the current study was not to demonstrate another replication of our original finding (Kindt et al., 2009), we did not include a control condition. Nonetheless, the data clearly show that propranolol HCl before memory reactivation did at least not erase the emotional expression of fear memory as we have reliably demonstrated in a series of studies (Kindt et al., 2009; Soeter and Kindt, 2010, 2011a, 2012a,b; Sevenster et al., 2012, 2013, 2014a)^2^.

To conclude, fear extinction is a very effective and well-established strategy to reduce conditioned fear responding but the fear memory remains available after successful extinction, which might eventually result in the return of fear (Bouton, 2002). An alternative strategy to persistently reduce learned fear responding is disrupting memory reconsolidation with the promise to provide long-term cure for patients with psychiatric conditions, like PTSD and drug addiction. Notwithstanding the robustness of these fear-reducing strategies, we currently showed that the prerequisites for triggering memory reconsolidation or extinction learning can be difficult and are not automatically obtained.

## Conflict of Interest Statement

The authors declare that the research was conducted in the absence of any commercial or financial relationships that could be construed as a potential conflict of interest.

## Footnotes

^1^Note that the current study as well as the study of Sevenster et al. (2012) included all trials in the analysis, whereas the other studies compared the start versus the end of acquisition. Nevertheless, re-analysis of acquisition (start versus end) still showed lower effect sizes (acquisition current study: CS × Trial: *F* (1,43) = 12.38, *p* = 0.001, η_p_2 = 0.22 versus acquisition Kindt et al. (2009): CS × Trial: *F* (1,38) = 46.91, *p <* 0.001, η_p_2 = 0.55).

^2^Note that the reported effect sizes of our previous studies, which compared a propranolol HCl memory reactivation condition with a control condition on fearful responding (i.e., startle potentiation) at day 3, were medium to large (cf. Cohen, 1988). Additionally, we calculated the Bayesian Factor (BF) for two of our previous studies (Kindt et al., 2009; Soeter and Kindt, 2010) where the same experimental design for the Propranolol HCl condition was used as in the current study. BF is a measure for the probability of the data under one hypothesis relative to the other hypothesis (i.e., null hypothesis versus alternative hypothesis or vice versa). The BF was calculated for the observed absence (i.e., null result) of a differential startle response between CS1 and CS2 at the first trials of extinction training at day 3 in our previous data. We used an online program (http://pcl.missouri.edu) to calculate the BF as a result of a default Bayesian *t*-test (for details, see Rouder et al., 2009). The BF_0A_ favored in both studies the null hypothesis over the alternative hypothesis (Kindt et al., 2009: BF_0A_ = 2.93; Soeter and Kindt, 2010: BF_0A_ = 2.97).

## References

[B1] Agren T. Furmark T. Eriksson E. Fredrikson M. (2012). Human fear reconsolidation and allelic differences in serotonergic and dopaminergic genes. Transl. Psychiatry 2, e76. 10.1038/tp.2012.5 22832813 PMC3309551

[B2] Blumenthal T. D. Cuthbert B. N. Filion D. L. Hackley S. Lipp O. V. Van Boxtel A. (2005). Committee report: guidelines for human startle eyeblink electromyographic studies. Psychophysiology 42, 1–15. 10.1111/j.1469-8986.2005.00271.x 15720576

[B3] Bos M. G. N. Beckers T. Kindt M. (2012). The effects of noradrenergic blockade on extinction in humans. Biol. Psychol. 89, 598–605. 10.1016/j.biopsycho.2012.01.007 22321909

[B4] Bouton M. E. (2002). Context, ambiguity, and unlearning: sources of relapse after behavioral extinction. Biol. Psychiatry 52, 976–986. 10.1016/S0006-3223(02)01546-9 12437938

[B5] Cohen J. (1988). Statistical Power Analysis for the Behavioral Sciences. Hillsdale, NJ: Erlbaum.

[B6] Díaz-Mataix L. Ruiz Martinez R. C. Schafe G. E. Ledoux J. E. Doyère V. (2013). Detection of a temporal error triggers reconsolidation of amygdala-dependent memories. Curr. Biol. 23, 467–472. 10.1016/j.cub.2013.01.053 23453952 PMC3606686

[B7] Eisenberg M. Kobilo T. Berman D. E. Dudai Y. (2003). Stability of retrieved memory: inverse correlation with trace dominance. Science 301, 1102–1104. 10.1126/science.1086881 12934010

[B8] Field A. (2013). Discovering Statistics Using IBM SPSS Statistics. London: Sage Publications Limited.

[B9] Forcato C. Argibay P. Pedreira M. Maldonado H. (2009). Human reconsolidation does not always occur when a memory is retrieved: the relevance of the reminder structure. Neurobiol. Learn. Mem. 91, 50–57. 10.1016/j.nlm.2008.09.011 18854218

[B10] Forcato C. Rodríguez M. L. C. Pedreira M. E. Maldonado H. (2010). Reconsolidation in humans opens up declarative memory to the entrance of new information. Neurobiol. Learn. Mem. 93, 77–84. 10.1016/j.nlm.2009.08.006 19703575

[B11] Fowles D. C. Christie M. J. Edelberg R. Grings W. W. Lykken D. T. Venables P. H. (1981). Publication recommendations for electrodermal measurements. Psychophysiology 18, 232–239 10.1111/j.1469-8986.1981.tb03024.x 7291438

[B12] Gilman A. G. Goodman L. S. (1996). Goodman and Gilman’s the Pharmacological Basis of Therapeutics. New York: McGraw-Hill.

[B13] Grillon C. Cordova J. Morgan C. A. III Charney D. S. Davis M. (2004). Effects of the beta-blocker propranolol on cued and contextual fear conditioning in humans. Psychopharmacology 175, 342–352. 10.1007/s00213-004-1819-5 15007536

[B14] Hamm A. O. Weike A. I. (2005). The neuropsychology of learning and fear regulations. Int. J. Psychophysiol. 57, 5–14 10.1016/j.ijpsycho.2005.01.006 15935258

[B15] Hupbach A. Hardt O. Gomez R. Nadel L. (2008). The dynamics of memory: context-dependent updating. Learn. Mem. 15, 574–579. 10.1101/lm.1022308 18685148

[B16] Kindt M. Soeter M. Vervliet B. (2009). Beyond extinction: erasing human fear responses and preventing the return of fear. Nat. Neurosci. 12, 256–258. 10.1038/nn.2271 19219038

[B17] Klorman R. Weerts T. C. Hastings J. E. Melamed B. G. Lang P. J. (1974). Psychometric description of some specific-fear questionnaires. Behav. Ther. 5, 401–409 10.1016/S0005-7894(74)80008-0

[B18] Lang P. J. Bradley M. M. Cuthbert B. N. (2005). International Affective Picture System (IAPS): Affective Ratings of Pictures and Instruction Manual. Gainsville, FL: University of Florida.

[B19] Lee J. L. C. (2009). Reconsolidation: maintaining memory relevance. Trends Neurosci. 32, 413–420. 10.1016/j.tins.2009.05.002 19640595 PMC3650827

[B20] Milekic M. H. Alberini C. M. (2002). Temporally graded requirement for protein synthesis following memory reactivation. Neuron 36, 521–525. 10.1016/S0896-6273(02)00976-5 12408853

[B21] Nader K. Hardt O. (2009). A single standard for memory: the case for reconsolidation. Nat. Rev. Neurosci. 10, 224–234. 10.1038/nrn2590 19229241

[B22] Nader K. Schafe G. E. Le Doux J. E. (2000). Fear memories require protein synthesis in the amygdala for reconsolidation after retrieval. Nature 406, 722–726. 10.1038/35021052 10963596

[B23] Pedreira M. E. Pérez-Cuesta L. M. Maldonado H. (2004). Mismatch between what is expected and what actually occurs triggers memory reconsolidation or extinction. Learn. Mem. 11, 579–585. 10.1101/lm.76904 15466312 PMC523076

[B24] Peterson R. A. Reiss S. (1992). Anxiety Sensitivity Index Manual. Worthington: International Diagnostic System.

[B25] Rouder J. N. Speckman P. L. Sun D. Morey R. D. Iverson G. (2009). Bayesian *t* test for accepting and rejecting the null hypothesis. Psychon Bull Rev 16, 225–237. 10.3758/PBR.16.2.225 19293088

[B26] Sara S. J. (2000). Retrieval and reconsolidation: toward a neurobiology of remembering. Learn. Mem. 7, 73–84 10.1101/lm.7.2.73 10753974

[B27] Sevenster D. Beckers T. Kindt M. (2012). Retrieval per se is not sufficient to trigger reconsolidation of human fear memory. Neurobiol. Learn. Mem. 97, 338–345. 10.1016/j.nlm.2012.01.009 22406658

[B28] Sevenster D. Beckers T. Kindt M. (2013). Prediction error governs pharmacologically induced amnesia for learned fear. Science 339, 830–833. 10.1126/science.1231357 23413355

[B29] Sevenster D. Beckers T. Kindt M. (2014a). Prediction error demarcates the transition from retrieval, to reconsolidation, to new learning. Learn. Mem. 21, 580–584. 10.1101/lm.035493.114 25320349 PMC4201815

[B30] Sevenster D. Beckers T. Kindt M. (2014b). Fear conditioning of SCR but not the startle reflex requires conscious discrimination of threat and safety. Front. Behav. Neurosci. 8:32. 10.3389/fnbeh.2014.00032 24616672 PMC3937874

[B31] Soeter M. Kindt M. (2010). Dissociating response systems: erasing fear from memory. Neurobiol. Learn. Mem. 94, 30–41. 10.1016/j.nlm.2010.03.004 20381628

[B32] Soeter M. Kindt M. (2011a). Disrupting reconsolidation: pharmacological and behavioral manipulations. Learn. Mem. 18, 357–366. 10.1101/lm.2148511 21576515

[B33] Soeter M. Kindt M. (2011b). Noradrenergic enhancement of associative fear memory in humans. Neurobiol. Learn. Mem. 96, 263–271. 10.1016/j.nlm.2011.05.003 21624479

[B34] Soeter M. Kindt M. (2012a). Erasing fear for an imagined threat event. Psychoneuroendocrinology 37, 1769–1779. 10.1016/j.psyneuen.2012.03.011 22503387

[B35] Soeter M. Kindt M. (2012b). Stimulation of the noradrenergic system during memory formation impairs extinction learning but not the disruption of reconsolidation. Neuropsychopharmacology 37, 1204–1213. 10.1038/npp.2011.307 22169947 PMC3306881

[B36] Soeter M. Kindt M. (2013). High trait anxiety: a challenge for disrupting fear memory reconsolidation. PLoS ONE 8:e75239. 10.1371/journal.pone.0075239 24260096 PMC3832500

[B37] Spielberger C. D. (1970). Manual for the State-Trait Anxiety Inventory. Palo Alto, CA: Consulting Psychologist Press.

[B38] Suzuki A. Josselyn S. A. Frankland P. W. Masushige S. Silva A. J. Kida S. (2004). Memory reconsolidation and extinction have distinct temporal and biochemical signatures. J. Neurosci. 24, 4787–4795. 10.1523/JNEUROSCI.5491-03.2004 15152039 PMC6729467

[B39] Van Boxtel A. Boelhouwer A. J. W. Bos A. R. (1998). Optimal EMG signal bandwidth and interelectrode distance for the recording of acoustic, electrocutaneous, and photic blink reflexes. Psychophysiology 35, 690–697. 10.1111/1469-8986.3560690 9844430

[B40] Wang S. de Oliveira Alvares L. Nader K. (2009). Cellular and systems mechanisms of memory strength as a constraint on auditory fear reconsolidation. Nat. Neurosci. 12, 905–912. 10.1038/nn.2350 19543280

